# Diode laser transscleral cyclophotocoagulation for uveitis-glaucoma-hyphema syndrome: A case report

**DOI:** 10.1097/MD.0000000000018637

**Published:** 2020-02-14

**Authors:** Zhengfeng Liu, Feng Zhang, Ying Wen, Xiujuan Du, Xuemei Pan, Hongsheng Bi

**Affiliations:** a Medical School of Ophthalmology & Optometry, Shandong University of Traditional Chinese Medicine; b Shandong Provincial Hospital Affiliated to Shandong University; c Shandong Maternity & Child Health Care Hospital; d Affiliated Eye Hospital of Shandong University of Traditional Chinese Medicine, Jinan 250002, China.

**Keywords:** acrylic single-piece intraocular lens, case report, diode laser transscleral cyclophotocoagulation, uveitis-glaucoma-hyphema (UGH) syndrome

## Abstract

**Rationale:**

To report a case of diode laser transscleral cyclophotocoagulation (DLTSC) for uveitis-glaucoma-hyphema syndrome (UGH).

**Patient concerns:**

The patient developed UGH on the right eye (OD) after vitrectomy and intraocular lens (IOL) implantation.

**Diagnoses:**

Best corrected visual acuity (BCVA) was HM/50 cm, intraocular pressure (IOP) was 51.3 mm Hg on the OD. He was found to have 3+ anterior chamber cells. A B-scan ultrasound showed vitreous opacity. Ultrasound biomicroscopy (UBM) showed the chafing between the IOL and the posterior surface of the iris. Thus, he was diagnosed as UGH on the OD.

**Interventions:**

The patient was worried about the complications for removal of the IOL, a DLTSC approach was performed.

**Outcomes:**

BCVA was 20/40 on the OD, IOP was 12 mm Hg on the OD. There were no anterior chamber inflammation and no vitreous opacity. UBM showed there was no contact between IOL and the posterior surface of the iris, the fundus of the eye was clearly visible.

**Lessons:**

UGH syndrome is a severe complication of cataract extraction. IOL extraction has been the traditional approach to treatment. DLTSC can be an option when the IOL is slightly tilted.

## 1 Introduction

Uveitis-glaucoma-hyphema (UGH) syndrome is a severe complication of cataract extraction and a cause for blurry vision weeks to months after surgery. UGH syndrome is classically related with anterior chamber intraocular lens (IOL).^[1]^ However, there are reports cases of decentred or dislocated posterior chamber IOL/capsular bag complex as possible triggers.^[2,3]^ Explantation of the implant has been the traditional approach to treatment.^[4]^ Zhang et al reported a case of UGH relieved areas of chafing and resolved symptoms by endoscopic cyclophotocoagulation.^[5]^ Here, a case of UGH treated by diode laser transscleral cyclophotocoagulation (DLTSC).

## 2 Case presentation

**Figure 1:**
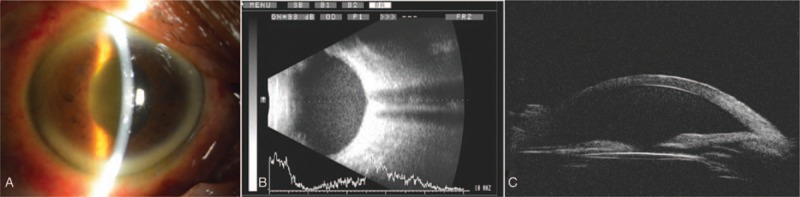
Slit-lamp examination (A) showed 3+ anterior chamber cells on OD. B-scan ultrasound (B) showed vitreous opacity. UBM (C) showed the chafing between the IOL and the posterior surface of the iris at 5 o’clock when the eye moved. IOL = intraocular lens, OD = right eye, UBM = ultrasound biomicroscopy.

**Figure 2:**
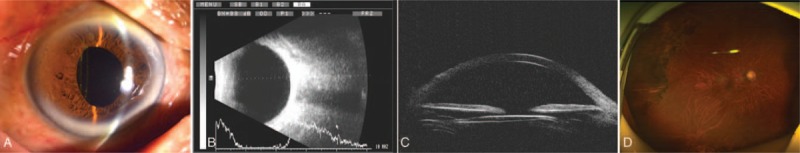
Slit-lamp examination (A) showed no anterior chamber inflammation. B-scan ultrasound (B) showed no vitreous opacity. UBM (C) showed there was no contact between IOL and the posterior surface of the iris, the fundus of the eye was clearly visible (D). IOL = intraocular lens, UBM = ultrasound biomicroscopy.

A male first presented to our hospital in June 2010 for his rhegmatogenous retinal detachment on the right eye (OD) when he was 58-year-old. Vitrectomy was performed and perfluocarbon gas was filled. One year later, phacoemulsification was performed because of cataract on the OD. Cataract surgery was uneventful and a monofocal single-piece IOL (+19.5D, NATURAL, Alcon) was implanted in the capsular bag. Postoperative best corrected visual acuity (BCVA) was 20/25 on the OD. However, the patient did not have further consultation because of his busy gardening work. Now, the patient complained of distending pain of eye and blurry vision over a period of half a year on the OD. BCVA was HM/50 cm OD and 20/25 on the left eye (OS). Intraocular pressure (IOP) was 51.3 mm Hg OD and 19.0 mm Hg OS. On examination, she was found to have 3+ anterior chamber cells on OD (Fig. 1A). A B-scan ultrasound showed vitreous opacity (Fig. 1B). Ultrasound biomicroscopy (UBM) showed the chafing between the IOL and the posterior surface of the iris at 5-o’clock (Fig. 1C) when the eye moved. Also, both his erythrocyte sedimentation rate and serum C-reactive protein concentration were normal. No abnormalities were found in systemic immunity and virus series tests. The patient was diagnosed with UGH syndrome. Medical management was been done first. However, neither IOP nor inflammation could be controlled. Removal of the IOL in this case was an unattractive option because the patient was worried about risks of surgical complications, a DLTSC approach was performed. The DLTSC was with the OcuLight SLx 810 nm diode laser photocoagulator and the handheld fiberoptic G-probe. The eye was performed under local anesthesia (2 ml of 2% lidocaine and 2 ml 1.5% ropivacaine as a retrobulbar injection). The laser was set at an initial power of 1750 mW and a duration of 2 seconds. The laser power was to achieve a ‘burst’ sound in roughly half of the laser applications. Laser applications were spaced evenly over the inferior 180 degrees, while sparing the 4- and 6-o’clock regions, all 15 light condensation points. The patient was not uncomfortable, no bleeding in the eye, and safe to return to the ward. After 12 months of follow-up, BCVA was 20/40 OD and 20/25 OS. IOP was 12 mm Hg OD and 15 mm Hg OS. There were no anterior chamber inflammation (Fig. 2A), no vitreous opacity (Fig. 2B). UBM showed there was no contact between IOL and the posterior surface of the iris (Fig. 2C), the fundus of the eye was clearly visible (Fig. 2D).

## 3 Discussion

The main cause of UGH syndrome is mechanical chafing of uveal structures caused by IOL,^[6]^ resulting in the breakdown of the blood-aqueous barrier and enabling cytokines to trigger an inflammatory cascade, causing a chronic inflammation as well as recurrent hyphema or microhyphemas and glaucoma.^[7]^

In patients with UGH syndrome, topical and systemic medication (corticosteroids along with IOP lowering medication) control the anterior inflammation, reduce the IOP and bring symptomatic relief in the short term. Explantation exchange, new treatment options can be the placement of a capsular tension ring to redistribute zonular tension^[8]^ and anti-vascular endothelial growth factor (anti-VEGF) therapy,^[9]^ and local laser iridoplasty,^[10]^ and IOL suturing to the iris,^[11]^ focal endoscopic cyclophotocoagulation.^[5]^

In the present case, the patient often bowed his head at work and caused chafed between the iris and the IOL. Because the patient had undergone vitrectomy, it made the chafe worse. Explantation of the IOL was discussed, the patient refused because he was worried about the risk of surgery. According to the UBM, the DLTSC was performed. The laser shrinks the ciliary processes, which may reduce the iris contact on the IOL, thus relieve the chafe. DLTSC is traditionally used only for late glaucoma, with little or no visual potential. It has become a minimally invasive treatment for glaucoma.^[12]^ Potential complications of anti-VEGF therapy include vision loss, uveitis, hypotony, and rarely atrophy of eyeball.^[13–15]^ To avoid the complications, we photocoagulated at 4- and 6-o’clock regions and only 15 points. And, good results have been received.

In conclusion, we present a case of UGH syndrome controlled with DLTS. Compared with other operations, DLTS has minimal damage and no risk of infections. However, severe titled IOL is not applicable. Another, the chafing between the IOL and the posterior surface of the iris is determined by UBM when the DLTS is performed. Despite potential side effects, DLTS offers an alternative therapy option to UGH syndrome when the IOL is slightly tilted.

## Author contributions

**Data curation:** Zhengfeng Liu.

**Investigation:** Xiujuan Du.

**Project administration:** Xuemei Pan.

**Writing – Original Draft:** Zhengfeng Liu, Feng Zhang.

**Writing – Review & Editing:** Feng Zhang, Hongsheng Bi, Xuemei Pan.
